# Blood toxicogenomics reveals potential biomarkers for management of idiosyncratic drug-induced liver injury

**DOI:** 10.3389/fgene.2025.1524433

**Published:** 2025-03-25

**Authors:** Rachel J. Church, Benedict Anchang, Brian D. Bennett, Pierre R. Bushel, Paul B. Watkins

**Affiliations:** ^1^ Division of Pharmacotherapy and Experimental Therapeutics, University of North Carolina Eshelman School of Pharmacy, Chapel Hill, NC, United States; ^2^ Biostatistics and Computational Biology Branch, National Institute of Environmental Health Sciences, Research Triangle Park, NC, United States; ^3^ Integrative Bioinformatics Group, National Institute of Environmental Health Sciences, Research Triangle Park, NC, United States

**Keywords:** drug-induced liver injury, hepatotoxicity, RNA-seq, immune-regulated pathways, peripheral blood transcriptomics, differentially expressed genes (DEGs)

## Abstract

**Introduction:** Accurate diagnosis, assessment, and prognosis of idiosyncratic drug-induced liver injury (IDILI) is problematic, in part due to the shortcomings of traditional blood biomarkers. Studies in rodents and healthy volunteers have supported that RNA transcript changes in whole blood may address some of these shortcomings.

**Methods:** In this study, we conducted RNA-Seq analysis on peripheral blood samples collected from 55 patients with acute IDILI and 17 patients with liver injuries not attributed to IDILI.

**Results and discussion:** Three differentially expressed genes (DEGs; *CFD*, *SQLE*, and *INKA1*) were identified as significantly associated with IDILI vs. other liver injuries. No DEGs were identified comparing IDILI patients to the 5 patients with autoimmune hepatitis, suggesting possible common underlying mechanisms. Two genes (*VMO1* and *EFNA1*) were significantly associated with hepatocellular injury compared to mixed/cholestatic injury. When patients with severe vs. milder IDILI were compared, we identified over 500 DEGs. The top pathways identified from these DEGs had in common down regulation of multiple T-cell specific genes. Further analyses confirmed that these changes could largely be accounted for by a fall in the concentration of circulating T-cells during severe DILI, perhaps due to exhaustion or sequestration of these cells in the liver. Exploration of DEGs specific for the individual causal agents was largely unsuccessful, but isoniazid-induced IDILI demonstrated 25 DEGs compared to other non-isoniazid IDILI cases. Finally, among the 14 IDILI patients that had hepatocellular jaundice (i.e., Hy’s Law cases), we identified 39 DEGs between the 4 patients with fatal or liver transplantation outcomes compared to those that recovered. These findings suggest the potential for blood-based transcriptomic biomarkers to aid in the diagnosis and prognostic stratification of IDILI.

## 1 Introduction

Drug-induced liver injury (DILI) caused by small molecule drugs, biologics, or herbal/dietary supplements is an ongoing concern for patients, clinicians, drug developers, and regulatory authorities. Some compounds, like acetaminophen, provoke a predictable, dose-dependent DILI (“intrinsic” DILI). Drugs capable of intrinsic DILI can generally be safely administered to patients as long as administered doses are below the toxic threshold. However, the majority of DILI-eliciting therapeutics cause idiosyncratic liver injury (IDILI). Drugs that can cause IDILI are safe for the vast majority of treated patients receiving therapeutic dosing, but clinically important and sometimes life-threatening liver injury can rarely occur, typically in fewer than 1 in 5,000 treated patients (Watkins, 2019). IDILI liability in a new drug candidate often goes undetected until late clinical development or may only become evident after a new drug has entered the market when a very large number of patients are exposed (Chalasani et al., 2025).

While most IDILI patients recover following discontinuation of the offending drug, data collected from the US Drug Induced Liver Injury Network (DILIN) show up to 20% of IDILI patients may have ongoing injury 6 months after the onset of the event (“chronic” injury) and 75% of these patients may still have ongoing injury after 1 year (Fontana et al., 2015). The long-term outcome of this chronic injury is unclear. Of greater concern, data from large registries indicate that around 10% of IDILI patients will either die or require a liver transplant (Garcia-Cortes et al., 2020).

Detection and assessment of the severity of IDILI primarily relies on serum levels of biomarkers that have been in use for over half a century: alanine aminotransferase (ALT), aspartate aminotransferase (AST), alkaline phosphatase (ALP), and bilirubin. But elevations in serum levels of these biomarkers simply indicate that liver injury is present and are not useful in distinguishing IDILI from the many other potential causes of liver injury, including viral and autoimmune hepatitis. Furthermore, the pattern of abnormalities in these serum biomarkers do not reliably help identify the specific drug causing IDILI in the patient receiving polypharmacy. For this reason, physicians may be compelled to stop treatment with multiple drugs in patients experiencing IDILI, potentially leaving underlying diseases sub-optimally treated (Bjornsson et al., 2010; Teschke et al., 2014; Watkins, 2015). Furthermore, there are some drugs that at therapeutic doses can cause frequent and marked elevations in the traditional serum biomarkers yet these drugs do not have substantial liver safety concerns (Watkins et al., 1994; Harrill et al., 2012; Singhal et al., 2014). There has therefore been intense scientific interest in discovery of new liver safety biomarkers, including analyses of serum microRNAs (Russo et al., 2017) and proteins (Church et al., 2019). To date, this research has not produced novel liver safety biomarkers accepted into clinical or drug development practice.

Established in 2004, the DILIN created a registry and tissue bank from patients who have experienced IDILI. Because patients are often referred to the DILIN enrollment centers from outside healthcare providers, and because patients were initially not enrolled into the DILIN registry until all other potential causes of liver injury were excluded, the IDILI event may have largely or completely resolved at the time of enrollment and biospecimen collection. In 2014 a new Acute Protocol was initiated for IDILI patients enrolled within 2 weeks of recognition of liver injury and prior to diagnosis of IDILI, and who at the time of enrollment had biochemical evidence of ongoing liver injury. In addition to the usual collection of DNA, serum and plasma, whole blood was collected in PAXgene tubes to enable toxicogenomic analysis. Preclinical and clinical studies of acetaminophen toxicity have demonstrated that blood toxicogenomics can reveal novel candidate genes and pathways which may inform mechanisms underlying liver responses (Bushel et al., 2007; Huang et al., 2010; Zhang et al., 2012; Fannin et al., 2016; Bushel et al., 2017; Lauschke, 2021; Segovia-Zafra et al., 2021). For instance, serial blood toxicogenomic analysis was assessed in healthy adult volunteers receiving recurrent therapeutic doses of acetaminophen and some of the subjects experienced substantial elevations in serum ALT. Those volunteers who did not experience ALT elevations showed a blood toxicogenomic pattern consistent with immune tolerance, whereas those that experienced serum ALT elevations showed changes consistent with pro-inflammatory signaling (Fannin et al., 2016). It is now appreciated that IDILI often involves immune responses, including immune tolerance (Mosedale and Watkins, 2020; Allison et al., 2023), supporting the possibility that blood toxicogenomics might reveal useful new biomarkers for IDILI. We report here the results of transcript profiling of whole blood obtained from the first 78 patients entering the DILIN Acute Protocol.

## 2 Materials and methods

### 2.1 DILIN subjects

All subjects in this study were enrolled in a registry and biobank of DILIN. DILIN prospectively collects clinical data along with biological samples from patients who come to medical attention due to IDILI within 6 months of IDILI onset. The criteria for enrollment into DILIN have been described previously (Fontana et al., 2014). In the Acute Protocol initiated in 2014 patients enrolled within 2 weeks of liver injury detection had whole blood samples collected into PAXgene tubes, with total RNA subsequently isolated and stored at −80°C. In this study, samples from all 78 DILIN subjects that met RNA-sequencing requirements (total RNA of ≥0.5 µg in a concentration of at least 10 μg/mL) were utilized. Causality assessment was performed according the DILIN process (Fontana et al., 2009). Because enrollment occurred prior to confident diagnosis of IDILI, some enrolled patients were determined to have less than 50% chance of IDILI (i.e., “unlikely” or “possible” causality scores) but most were assessed as having ≥50% chance of IDILI (i.e., “probable,” “highly likely,” and “definite” scores). The pattern of IDILI injury was determined based on the “R” ratio of serum ALT to ALP values collected at the first visit (expressed as a multiple of the ULN). An R value ≥5 indicates hepatocellular injury while an R value ≤2 indicates cholestatic injury. R values between 2 and 5 are considered “mixed” injury (Aithal et al., 2011). Patients’ consented to having their information used and DILIN approved the use of patient information for this study.

### 2.2 RNA sequencing and data processing

**FIGURE 1:**
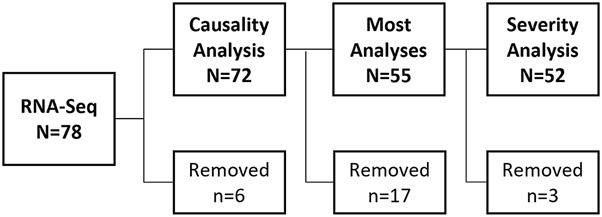
Samples used for DILIN RNA-Seq Analyses. Whole blood from N = 78 DILIN patients were RNA-Sequenced. Following sequencing and prior to any data analyses, N = 6 samples were removed (N = 1 sample had low read counts, N = 3 samples had unique population and ethnicity combinations, N = 1 processed in separate batch, and N = 1 was an outlier by PCA analysis). A causality analysis was performed using data from the remaining N = 72 patients. Most subsequent analyses were performed using only data from patients with a “high causality” score (≥50% likely that the liver injury was caused by use of indicated drug) removing data from the N = 17 patients with a “low” causality score (<50% chance liver injury was caused by a drug). Expert review of the remaining cases (by author PBW) identified N = 3 patients where severity score could not be determined due confounding medical conditions; therefore these patients were removed from analyses examining gene differences related to IDILI severity.

RNA-sequencing was performed on samples from 78 DILIN patients. Figure 1 outlines the number of samples removed or moved forward in each analysis step. Single-endRNA sequencing was conducted with a read length of 51 nucleotides and an average read-depth of approximately 40 million reads per sample. Following sequencing, one sample was excluded due to very low read counts (<14,000 read counts) leaving 77 samples for further analysis. The remaining samples were aligned to the hg38 reference genome using the “Spliced Transcripts Alignment to a Reference” (STAR) tool v2.6.0c (Dobin et al., 2013). Counts were quantitated and summarized with featureCounts (Liao et al., 2014) from the Subread package v1.5.1 (Liao et al., 2013) using GENCODE version 38 (Frankish et al., 2023). The data used in this study has been deposited under the Gene Expression Omnibus (GEO) accession number GSE275008.

### 2.3 DESeq analysis

Initial pair-wise data analyses of samples were performed using the DESeq2 R package v1.32.0 (Love et al., 2014). This tool fits a linear model for each gene using the coefficients described for each analysis (e.g., low causality score vs. high causality score). One coefficient is selected, and a p-value is calculated for each gene using the Wald test based on the significance of that coefficient. P-values were adjusted for multiple testing using the Benjamini and Hochberg false discovery rate procedure (Benjamini and Hochberg, 1995). The DESeq2 tool applies the “apeglm” LFC shrinkage algorithm and reports a corrected log2 normalized fold change (Zhu et al., 2019). DEGs were defined as fold change of ≥1.5 and a false discovery rate (FDR) value of <0.05.

In each analysis, sex and self-reported population and ethnicity combinations were used as random effects in the model to reduce the impact of these as confounding factors. Out of the 77 individuals with sufficient read counts, those in the population and ethnicity groups of “Afro-descendant population and non-Latino population,” “White population and Non-Latino population,” or “White population or Other population and Latino population” were used. Three individuals had unique population and ethnicity combinations (N = 1 “Asian population,” N = 1 “Other population and Non-Latino population,” and N = 1 “Afro-descendant population and Latino population”) and were removed from downstream analyses because estimating effects requires more than one individual per group. Another individual was removed because their sample was processed in a separate batch. Finally, one sample from a subject whose liver injury was adjudicated as being only “possibly” related to drug (25%–50% chance), with malignancy being listed as a more likely cause, was removed from analysis. This individual had an RNA profile that was considerably different from the others and was an outlier in PCA analysis (data not shown). After excluding these five individuals, the remaining N = 72 samples were used to identify differentially expressed genes (DEGs) associated with IDILI causality. One additional analysis compared all IDILI patients with a high causality score (N = 55) to patients with a low causality score that had an alternative diagnosis of autoimmune hepatitis (N = 5; Supplementary Table S1). All subsequent analyses included only data from patients with a high causality score. An additional N = 3 samples were removed (leaving N = 52) when DEGs related to IDILI severity were interrogated. Upon expert case review (author PBW), it was determined that all three had confounders making it impossible to assign a severity score to the IDILI event (Supplementary Table S2). For the DESeq severity analysis, patients with mild, moderate and moderate-hospitalized severity scores were combined into a “low severity” group while patients with severity scores of severe or fatal were combined into a “high severity.”

### 2.4 Ingenuity pathway analysis

To explore DEGs at the pathway level, a core analysis was run using the Ingenuity Pathway Analysis (IPA) software v76765844. This analysis focused on identifying altered canonical pathways using genes from our analyses that met the thresholding criteria. Pathway significance was determined using Fisher’s Exact test and a Benjamini-Hochberg test to correct for false discoveries.

### 2.5 CIBERSORTx analysis

Data analysis was performed using the CIBERSORTx web-based tool (Newman et al., 2019). This tool applies a deconvolution algorithm used to extract signals of interest from a mixture. Specifically, it takes expression data and attempts to estimate the percentage of member cell types for each sample based on a model built from a separate training data set with known cell types. CIBERSORTx is a robust method optimized for the identification of immune cell types (Alonso-Moreda et al., 2023). We utilized a data set built into the CIBERSORTx tool with immune cells (LM22) as a training expression data set. In this analysis, three groups were compared: “mild,” “moderate” (consisting of moderate and moderate hospitalized), and “severe” (consisting of severe and fatal cases). Absolute T cell concentration was estimated by multiplying the CIBERSORTx estimated total T cell percentage by the concentration of white blood cells measured in DILIN patients, for those that had a WBC count collected within 4 days of the date the PAXgene sample was collected (51 of the 52 subjects included in the severity analysis. Boxplots were generated using the “boxplot” function built into R v3.3.2. A one-way analysis of variance (ANOVA) model was utilized to determine whether there were significant differences across the groups. For each group, the Shapiro-Wilk test was used to test the normality of the data. The null-hypothesis is that the sample is normally distributed. Based on the p-values for all groups (p-values = 0.61, 0.14, and 0.11 for mild, moderate, and severe, respectively) we conclude that there isn’t sufficient evidence to suggest that the data is not normally distributed. The correlation between the fraction of lymphocyte values estimated by CIBERSORTx (including fractions for T cells, B cells, Plasma cells, and Natural Killer cells) and those observed clinically was determined using Pearson’s correlation (R). This analysis was conducted using N = 37 of 55 high causality subjects who had their fraction of lymphocyte values determined within 1 day of blood collection in PAXgene tubes.

## 3 Results

The 78 patient samples available from the DILIN Acute Protocol underwent RNAseq and 6 were excluded, leaving 72 for analyses (Figure 1).

### 3.1 Gene expression changes associated with IDILI causality

Because the blood PAXgene tubes were collected prior to establishing a diagnosis of IDILI, 17 patients out of the 72 were subsequently considered to most likely have causes of liver injury other than DILI. To determine if RNA expression in blood differs between patients with IDILI vs. patients with other causes of liver injury, we compared RNA-Seq data obtained from the 17 patients unlikely to have experienced IDILI to the data obtained in the 55 patients thought to probably have IDILI (here after referred to as IDILI patients; Table 1). Three DEGs passed our significance criteria for this analysis (Supplementary Table S3). Those DEGs are complement factor D (*CFD*), squalene epoxidase (*SQLE*), and inka box actin regulator 1 (*INKA1*).

**TABLE 1:** Demographic information of DILIN patients used to identify DEGs associated with IDILI.

DILIN patients (N = 72)		
	“Low” causality (N = 17)	“High” Causality (N = 55)
Age, y, median (min, max)	51.8 (26.0, 75.8)	56 (18, 88)
Sex, n (%)		
Female	10 (59)	26 (47)
Male	7 (41)	29 (53)
Population and ethnicity, n (%)		
Afro-descendant population and Non-Latino population	4 (24)	10 (18)
White population and Non-Latino population	11 (65)	34 (62)
White population or Other population and Latino population	2 (12)	11 (20)
Causality Score, n (%)		
Unlikely (<25%)	9 (53)	0 (0)
Possible (25%–50%)	8 (47)	0 (0)
Probable (50%–75%)	0 (0)	25 (45)
Highly Likely (75%–95%)	0 (0)	26 (47)
Definite (>95%)	0 (0)	4 (7)
Injury Severity Score, n (%)^a^		
Mild	3 (18)	10 (18)
Moderate	1 (6)	5 (9)
Moderate-Hospitalized	6 (35)	24 (44)
Severe	4 (24)	9 (13)
Fatal (liver transplant, death)	3 (18)	7 (16)
BMI, median (min, max)	29.9 (21.1, 43.7)	25.8 (18.2, 40.2)
Injury Pattern, n%		
Cholestatic/Mixed (R < 5)	8 (47)	29 (53)
Hepatocellular (R ≥ 5)	9 (53)	26 (47)
Hy’s Law		
Yes	NA	15
No	NA	40

^a^ Three high causality IDILI subjects listed in this table (n = 1 listed as having severe IDILI and n = 2 listed as having fatal IDILI) were removed from IDILI, severity analyses.

Because presentation of IDILI can sometimes mimic that of spontaneous autoimmune hepatitis (i.e., not drug-induced), we also determined whether any blood DEGs were observed between the 55 IDILI patients and the 5 patients given the alternate diagnosis of spontaneous autoimmune hepatitis (Supplementary Table S1). In this analysis, no genes were found to have an FDR value < 0.05 (Supplementary Table S4).

### 3.2 Gene expression changes associated IDILI phenotype

Among the 55 IDILI patients, 26 patients had hepatocellular injury (R ≥ 5) while 29 patients showed a mixed or cholestatic injury pattern (R < 5). R value is calculated as the value of serum ALT divided by the serum value of alkaline phosphatase (expressed as fold upper limits of normal) observed when the patient first qualified for enrollment in the DILIN. We explored blood DEGs between these groups and found that only two genes, vitelline membrane outer layer 1 homolog (*VMO1*) and ephrin A1 (*EFNA1*), passed our thresholding criteria with fold changes of 3.97 (FDR = 0.008) and 2.12 (FDR = 0.03), respectively, in hepatocellular injury. Refer to Supplementary Table S5 for the full analysis.

### 3.3 Gene expression changes associated with individual therapeutics

We next examined whether IDILI due to individual therapeutics were associated with specific blood gene expression changes. Amongst 55 IDILI patients, there were 32 unique therapeutics determined by the causality assessment process to be the primary causal IDILI agent (Supplementary Table S6). Of those, only four specific drugs were the causal agent in at least three subjects: amoxicillin/clavulanic acid (N = 4), atorvastatin (N = 3), cefazolin (N = 3), and isoniazid (INH; N = 3). In addition, various herbals and dietary supplements (HDS) were suspected to be the causal agent in 11 patients. Blood DEGs for patients in each of these five cohorts were individually compared against all other IDILI patients. Following the analysis, there were no DEGs that passed thresholding criteria for amoxicillin/clavulanic acid (Supplementary Table S7), atorvastatin (Supplementary Table S8), or the HDS products (Supplementary Table S9). Only one DEG, *RP3-465N24.5* (fold change = −3.81; FDR value = 0.01), passed thresholding criteria in patients with cefazolin-associated IDILI (Supplementary Table S10). In contrast, we observed N = 25 DEGs in subjects with INH-associated IDILI (Table 2; Supplementary Table S11). To determine if these DEGs represented changes in specific functional pathways, we conducted IPA pathway analysis; however, no pathways passed multiple test correction in this analysis.

**TABLE 2:** Gene expression changes related to Isoniazid IDILI Passing Thresholding Criteria.

Gene Symbol	Gene name	Fold change	FDR
*GTSF1*	gametocyte specific factor 1	−3.50	0.017
*TRBV7-2*	T cell receptor beta variable 7–2	−3.27	0.024
*BTBD11*	BTB domain containing 11	−2.32	0.031
*ZNF880*	zinc finger protein 880	−2.26	0.024
*MITD1*	microtubule interacting and trafficking domain containing 1	−1.60	0.022
*TMEM192*	transmembrane protein 192	1.61	0.017
*MAP3K8*	mitogen-activated protein kinase 8	1.75	0.034
*LPCAT3*	lysophosphatidylcholine acyltransferase 3	1.93	0.017
*CD44*	CD44 molecule (Indian blood group)	1.96	0.017
*TIAM2*	TIAM Rac1 associated GEF 2	2.04	0.022
*DCPS*	decapping enzyme, scavenger	2.32	0.009
*RP3-466P17.4*		2.83	0.017
*MIR124-1HG*	MIR124-1 host gene	3.07	0.017
*GYG1*	glycogenin 1	3.33	0.017
*CTB-131B5.2*		3.41	0.036
*CTC-232P5.3*		3.46	0.017
*EDNRB*	endothelin receptor type B	3.64	0.034
*PPARG*	peroxisome proliferator activated receptor gamma	3.65	0.034
*RD3L*	RD3 like	3.71	0.032
*AF064858.8*		3.87	0.022
*DLC1*	DLC1 Rho GTPase activating protein	3.90	0.015
*TDRD9*	tudor domain containing 9	3.92	0.015
*SEMA6B*	semaphorin 6B	4.04	0.014
*MYO10*	myosin X	4.14	0.014
*RNASE1*	ribonuclease A family member 1, pancreatic	4.39	0.014

Abbreviations: FDR, false discovery rate.

### 3.4 Gene expression changes associated with IDILI severity

**FIGURE 2:**
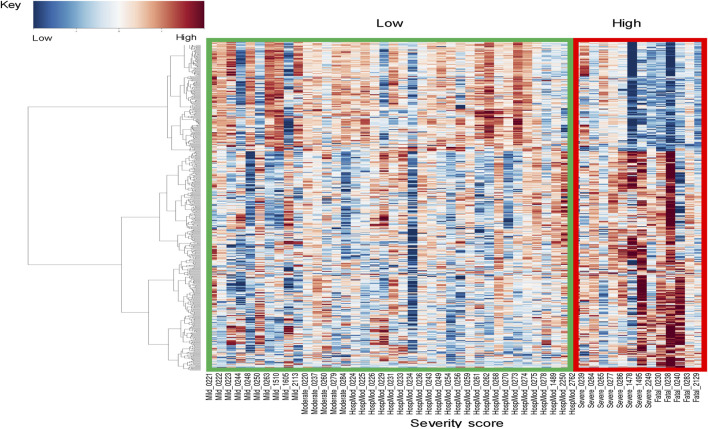
Gene expression profiles across DILIN severity. Heatmap of significant gene expression profiles for N = 52 patients, showing two major global signatures. To increase the power of differential analysis, patients with mild, moderate, and moderate-hospitalized severity scores are classified into a “low severity” group, while patients with severe or fatal scores are combined into a “high severity” group.

To explore whether severity of injury in IDILI patients was associated with gene expression changes in the blood, data from 3 patients were removed due to confounding events that prevented IDILI severity assessment (Supplementary Table S2). For this analysis, IDILI severity was assessed according to the DILIN criteria (Fontana et al., 2009). Patients with mild, moderate, or moderate-hospitalized severity (N = 39) were compared to patients with severe or fatal (N = 13) IDILI. Figure 2 shows the major gene expression patterns. We observed changes that passed thresholding criteria in 526 DEGs, representing reduced expression of 177 genes and 349 increased expression changes in more severe IDILI patients (Supplementary Table S12). The DEGs passing thresholding criteria were analyzed in IPA to determine canonical biological pathways altered in severe IDILI patients. The 10 most significantly enriched pathways were primarily related to immune cell regulation (Table 3). We noted that these pathways had similar numbers of increased and reduced DEGs represented from our dataset; therefore, we examined the individual DEGs in each of these pathways to determine how many unique genes were common across these pathways. We identified 6 unique DEGs that were elevated in at least one of the top 10 significant pathways (Supplementary Table S13) and 44 unique DEGs that were reduced across the same pathways (Supplementary Table S14). Interestingly, 37 of these reduced DEGs were represented in all 10 of the pathways. Of those, 36 were genes that encode T cell receptor (TCR) subunits. Furthermore, the remaining gene expressed in all 10 pathways *CD3d*, encodes a subunit of CD3, a T cell co-receptor. *CD28*, which encodes a protein expressed on T cells to provide a co- stimulatory signal for T cell activation, was represented in 5 of the 10 most significant canonical pathways. A list of all the pathways that passed multiple test correction can be found in Supplementary Table S15.

**TABLE 3:** Top ten most significant canonical pathways related to IDILI severity.

Caononical pathyway	FDR	Total molecules in pathway n	Significantly increased in pathway n (%)	Significantly reduced in pathway n (%)
1. Altered T Cell and B Cell Signaling in Rheumatoid Arthritis	8.64E-18	492	3 (0.6)	40 (8.1)
2. Th17 Activation Pathway	1.61E-17	484	3 (0.6)	39 (8.1)
3. Antiproliferative Role of TOB in T Cell Signaling	3.48E-17	426	1 (0.2)	38 (8.9)
4. CTLA4 Signaling in Cytotoxic T Lymphocytes	3.48E-17	478	0 (0.0)	41 (8.6)
5. T Helper Cell Differentiation	1.11E-16	471	2 (0.4)	38 (8.1)
6. Autoimmune Thyroid Disease Signaling	1.72E-16	454	1 (0.2)	38 (8.4)
7. Calcium-induced T Lymphocyte Apoptosis	2.12E-16	460	0 (0.0)	39 (8.5)
8. T Cell Exhaustion Signaling Pathway	2.12E-16	566	3 (0.5)	40 (7.1)
9. Lipid Antigen Presentation by CD1	2.80E-16	416	0 (0.0)	37 (8.9)
10. Hematopoiesis from Pluripotent Stem Cells	2.80E-16	442	1 (0.2)	37 (8.4)

Abbreviations: FDR, false discovery rate.

**FIGURE 3:**
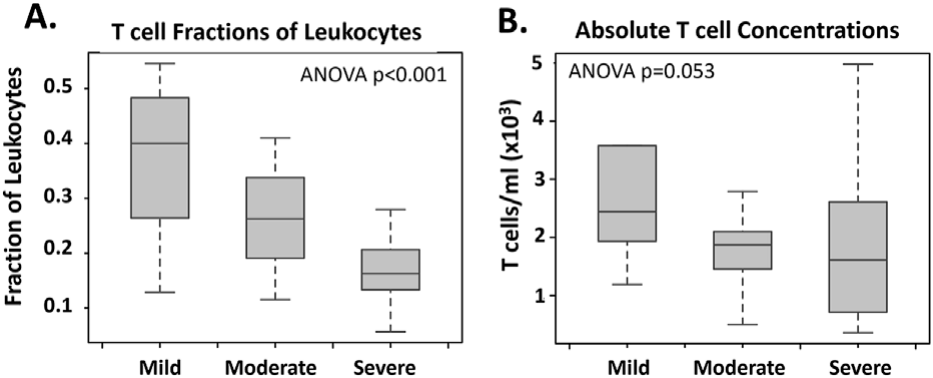
Total T Cell Changes Related to IDILI Severity CIBERSORTx analysis was utilized to determine total T cell fraction **(A)** changes related to injury severity in n = 52 high causality IDILI patients. Ordinal rankings of “mild” (mild severity score, n = 10), “moderate” (moderate and moderate-hospitalized severity scores n = 29), and “severe” (severe and fatal severity scores, n = 13) were assigned. A built-in immune cells (LM22) data set was utilized as a training expression data set. Total T cell concentration **(B)** was estimated in the n = 51 of these patients who had a blood sample for total WBC measurement collected within 4 days of RNA-seq blood sample collection (n = 1 moderate severity IDILI patient removed). The fraction of T cells determined using CIBERSORTx was multiplied by the WBC concentration for each patient to estimate the concentration of total cells. Significance was calculated using a one-way ANOVA test and was p < 0.001 and p = 0.053 for A and B, respectively.

We hypothesized that the reduced levels of DEGs related to T cell receptors in severe IDILI could be due to a reduced presence of T cells in the blood of these patients. To test this hypothesis, we conducted a CIBERSORTx analysis using a built-in immune cell training dataset. In this analysis, we observed a significant reduction in the predicted fraction of total T cells amongst all lymphocytes in the blood associated with IDILI severity (Figure 3A; ANOVA p < 0.001). This reduction could be due to a true reduction in T-cells in blood, or due to increased fraction of other white blood cell types in the circulation of patients with milder IDILI. In 51 of the 52 patients studied in our severity analysis, a differential white blood cell count had been collected within 4 days of the RNA-seq blood sample. In those patients, total T cell concentration was estimated (Figure 3B). Total T cell counts trended towards reduction with injury severity (ANOVA p = 0.053). As a proof of principle of the CIBERSORTx technique, we additionally explored the correlation between the CIBERSORTx predicted lymphocyte fraction and the observed lymphocyte fraction in a subset of IDILI subjects (N = 37 of 55) who had fraction lymphocytes measured in their blood within 1 day of study blood collection. In those subjects we observed a high correlation (r = 0.8406; p < 0.0001) between the fraction observed experimentally and the fractions predicted by CIBERSORTx (Supplementary Figure S1).

When the severity analysis was repeated with T cell percentage as a factor in the model, only four DEGs passed our thresholding criteria (Supplementary Table S16). Those DEGs were junctional adhesion molecule 3 (*JAM3*), fatty acid elongase 7 (*ELOVL7*), monocyte to macrophage differentiation associated (*MMD*), and RNA variant U1 small nuclear 2 (*RNVU1-2*).

### 3.5 Gene expression changes associated with chronic IDILI

Information related to IDILI recovery time was available for 47 of the N = 48 IDILI patients who did not experience fatal IDILI. We examined whether there were any DEGs between patients with acute IDILI (recovery occurred within 6 months of onset; N = 39) and those that experienced chronic IDILI (recovery time exceeded 6 months from onset; N = 8). In this analysis, no DEGs passed our thresholding criteria (Supplementary Table S17).

### 3.6 Gene expression changes associated with outcome in Hy’s Law patients

There were 14 IDILI patients in our cohort that met Hy’s Law criteria defined as hepatocellular injury (R-value >5.0 and a serum total bilirubin exceeding 2 times ULN at the time of blood sampling (2009 FDA guidance -(https://www.fda.gov/regulatory-information/search-fda-guidance-documents/drug-induced-liver-injury-premarketing-clinical-evaluation). One additional patient that met Hy’s Law criteria was excluded because confounding factors made it impossible to assign the appropriate severity category as described in Supplementary Table S2) We investigated whether there were gene expression differences between those patients who recovered (n = 10) and those that died due to the IDILI event (N = 4). In this analysis, 39 DEGs passed our thresholding criteria (Table 4; Supplementary Table S18), representing increased expression in 11 genes and reduced expression in 28 genes. When this DEG list was used to explore alterations in canonical biological pathways, no pathways passed multiple test correction.

**TABLE 4:** Gene expression changes between Hy’s Law patients who died (N = 4) and those that recovered (N = 10).

Gene Symbol	Gene name	Fold change	FDR
*TRAV26-2*	T cell receptor alpha variable 26–2	−12.97	0.0192
*SMTNL1*	smoothelin like 1	−5.11	0.0003
*NOS3*	nitric oxide synthase 3	−4.49	0.0111
*TRBV7-2*	T cell receptor beta variable 7–2	−3.81	0.0035
*IGLV3-21*	immunoglobulin lambda variable 3–21	−3.19	0.0131
*IGLC2*	immunoglobulin lambda constant 2	−2.98	0.0269
*WHRN*	whirlin	−2.94	0.0403
*LINC00649*	long intergenic non-protein coding RNA 649	−2.93	0.0078
*TRBV20-1*	T cell receptor beta variable 20–1	−2.70	0.0337
*PILRB*	paired immunoglobin like type 2 receptor beta	−2.46	0.0051
*AE000661.37*		−2.21	0.01917
*NCEH1*	neutral cholesterol ester hydrolase 1	1.81	0.0480
*TRPS1*	transcriptional repressor GATA binding 1	1.85	0.0267
*MS4A4E*	membrane spanning 4-domains A4E	2.07	0.0146
*VCAN-AS1*		2.23	0.0124
*MAFG*	MAF bZIP transcription factor G	2.36	0.0118
*MERTK*	MER proto-oncogene, tyrosine kinase	3.09	0.0480
*ACVRL1*	activin A receptor like type 1	3.18	0.0131
*FCAR*	Fc alpha receptor	3.61	0.0207
*GPNMB*	glycoprotein nmb	3.70	0.0118
*GFRA2*	GDNF family receptor alpha 2	3.94	0.0182
*ANKRD34B*	ankyrin repeat domain 34B	4.17	0.0118
*DLC1*	DLC1 Rho GTPase activating protein	4.42	0.0084
*COLEC12*	collectin subfamily member 12	4.52	0.0184
*SLC28A3*	solute carrier family 28 member 3	4.57	0.0084
*LPL*	lipoprotein lipase	4.76	0.0131
*ARHGAP29*	Rho GTPase activating protein 29	4.81	0.0054
*NDST3*	N-deacetylase and N-sulfotransferase 3	4.89	0.0131
*VSIG4*	V-set and immunoglobulin domain containing 4	5.04	0.0050
*SEMA6B*	semaphorin 6B	5.87	0.0010
*AF064858.8*		6.28	0.01585
*MYO10*	myosin X	6.30	0.0111
*TDRD9*	tudor domain containing 9	6.57	0.0003
*EDNRB*	endothelin receptor type B	7.49	0.0117
*RD3L*	RD3 like	8.74	0.0124
*MT-TL2*	tRNA	10.83	0.0182
*MT-ND6*	NADH dehydrogenase subunit 6	11.93	0.0009
*SLCO2B1*	solute carrier organic anion transporter family member 2B1	25.60	0.0003
*CCL24*	C-C motif chemokine ligand 24	71.10	0.0343

Abbreviations: FDR, false discovery rate.

## 4 Discussion

IDILI remains a serious concern for patients, clinicians, drug developers, and regulators largely due to the inadequacy of traditional biomarkers routinely used to detect and assess this affliction. Among other problems described elsewhere (Church et al., 2019), current biomarkers fail to differentiate IDILI from other forms of hepatic injury. In addition, current IDILI biomarkers have limited value in predicting outcome of an IDILI event, or in identifying the culprit agent in a patient receiving polypharmacy. These shortcomings can prevent clinicians from promptly taking appropriate therapeutic actions and may lead to the unnecessary discontinuation of multiple drugs that are safe and beneficial to the patient. While biopsies can often provide better characterization of liver injuries, liver biopsies have risks and are not routinely performed in the evaluation of acute liver injuries. There have been extensive investigations of blood in search of novel IDILI biomarkers that would address the shortcomings of traditional biomarkers, but none have yet been accepted into clinical practice.

Whole blood toxicogenomics has shown promise in providing mechanistic insight into multiple liver toxicities in rats and in acetaminophen liver reactions in healthy volunteers (Bushel et al., 2007; Mohr and Liew, 2007; Zhang et al., 2012; Bushel et al., 2017). The Acute Protocol in the DILIN network provided an opportunity to explore whether blood RNA profiles might yield biomarkers that could be helpful in the diagnosis, prognosis, and management of IDILI.

There are currently no biomarkers that can accurately diagnose IDILI as opposed to other liver injuries such as viral hepatitis and spontaneous autoimmune hepatitis. We therefore first examined whether unique DEGs in blood could differentiate individuals with IDILI (i.e., ≥50% likelihood of having IDILI) from those with more likely alternate diagnoses. In this analysis, the expression of only three genes was significantly altered in IDILI patients: *CFD*, *SQLE*, and *INKA1*. We observed an increase in *CFD* expression and a decrease in *SQLE* and *INKA1* expression in patients with IDILI. *CFD* encodes a protein, Complement Factor D, which is part of the alternative complement pathway, a component of the innate immune system. Mutations in this gene, resulting in reduced expression, have been associated with an increased susceptibility to bacterial infections (Sprong et al., 2006; Langereis et al., 2021). *SQLE* plays a key role in cholesterol metabolism and is highly upregulated in human Metabolic dysfunction Associated Steatohepatitis (MASH, formerly called NASH). *INKA1* is expressed in memory B-cells, naive B-cells, and basophils among immune cells. The functions of these three genes may suggest potential roles in IDILI pathogenesis.

We next examined whether there were gene expression differences associated with injury phenotype (hepatocellular vs. mixed/cholestatic) and found only VMO1 and EFNA1 passed thresholding criteria. Expression of these genes was elevated in patients with hepatocellular injury compared to patients with cholestatic or mixed pattern of injury. Little is known about the role of VMO1 in humans. One study examining gene expression in unique populations of human blood-derived monocytes found VMO1 to be highly expressed in non-classical (CD14^+^CD16^++^) monocytes (Wong et al., 2011). This class of monocyte was associated with a proapoptotic and antiproliferative state. EFNA1 is upregulated in multiple types of cancer, including hepatocellular carcinoma (Iida et al., 2005).

Even when biopsies can be performed, IDILI cannot be reliably distinguished from spontaneous autoimmune hepatitis (Bjornsson et al., 2010; Davern et al., 2011; Teschke et al., 2014). We therefore examined whether there were any significant DEGs between IDILI patients and the patients given the diagnosis of autoimmune hepatitis. Unfortunately, there were no DEGs that passed our thresholding criteria, which is suggestive of possible common underlying mechanisms.

The most remarkable finding from our study was the large number (>500) of DEGs associated with severe vs. milder forms of IDILI. This appeared to be largely accounted for by a reduction in expression of T-cell specific genes, which was closely associated with a fall in the fraction of T-cells among the total circulating white blood cell. Moreover, using the T-cell fraction as a covariate in the DEG model reduced the number of DEGs from >500 to just 4. It seems likely that in severe IDILI, T-cells have become exhausted and eliminated and/or are sequestered in the liver continuing to promote the severe injury. The fall in circulating T-cells may in part account for why it has been difficult to detect circulating T-cells reactive to the causal drug in IDILI (Whritenour et al., 2017))

Patients often take multiple medications and/or dietary supplements concurrently. Therefore, even when an IDILI diagnosis is suspected, clinicians may find it challenging to identify which drug the patient is taking is the offending agent. In our dataset, 4 single drugs caused IDILI in at least 3 patients and 11 patients had IDILI due to herbals or dietary supplements. In general, only INH-induced liver injury resulted in identification of significant DEGs compared to the subjects with IDILI not due to INH. Although no significant canonical pathways were observed, 25 genes passed our thresholding criteria in this analysis. The majority of these DEGs (80%) were elevated in patients with INH-induced liver injury. Notably, all three patients with INH-induced IDILI were amongst the “fatal” IDILI group. Specifically, two of these patients died because of their injury and one required a liver transplant. Of the 25 DEGs associated with INH-induced hepatotoxicity, only 10 (40%) overlapped with the DEGs related to IDILI severity discussed above. While one DEG *TRBV7-2,* encoding a TCR subunit was reduced in INH patients, it did not pass thresholding criteria in our severity analysis. Further, no significant differences in percentages or total concentration of circulating T cells were observed between INH-related injury patients and other patients with “severe” IDILI or all other IDILI patients (data not shown). It is therefore possible that at least some of the 25 DEGs associated with INH IDILI may be specific to this form of IDILI and not due to the severe nature of the events examined.

We also investigated DEGs in IDILI patients satisfying “Hy’s Law” who progressed to liver failure and death (or liver transplantation) vs. those who recovered. Hy’s Law criteria is defined as hepatocellular injury (R-value >5.0) and a serum total bilirubin exceeding 2 X ULN (2009 FDA guidance https://www.fda.gov/regulatory-information/search-fda-guidance-documents/drug-induced-liver-injury-premarketing-clinical-evaluation). Both the DILIN and European registries have reported that about 10% of IDILI patients who satisfy “Hy’s Law” will have a fatal outcome and it is not currently possible to accurately predict this outcome. We were therefore encouraged to identify 39 DEG’s between these two outcomes and, if validated in additional studies, monitoring some of these RNAs in blood may be useful in assessing prognosis in patients who satisfy Hy’s Law.

We acknowledge limitations in this study. The study utilized PAXgene tubes which primarily capture intracellular RNA, potentially missing important RNA changes occurring in extracellular. Additionally, we did not have access to whole blood RNA samples from individuals not experiencing liver injury. We pursued GTEX to obtain healthy control data, but these subjects were recently deceased and may not represent healthy controls. In addition, when we explored using these data as controls, we observed batch effects which would confound the analyses. The absence of healthy control data did not interfere with our ability to use DILIN data to investigate clinically important questions that are relevant to IDILI management (i.e., IDILI vs. other liver diseases, cholestatic/mixed vs. hepatocellular phenotype, and severity of injury). Finally, the relatively small number of samples, both in the dataset as a whole and in smaller subgroups, posed a challenge in identifying statistically significant changes and we did not have separate cohorts to validate what we did find. The authors anticipate that future studies with larger sample sizes and potentially more sensitive technologies may provide a more comprehensive understanding of the observed changes.

In summary, considering the limitations mentioned, the findings from this study suggest that blood-based transcriptomic biomarkers have potential to aid in the diagnosis and stratification of IDILI, particularly by identifying immune-regulated pathways.

## Data Availability

The datasets presented in this study can be found in online repositories. The names of the repository/repositories and accession number(s) can be found below: https://www.ncbi.nlm.nih.gov/geo/, GSE275008.
