# Supplementary material for: Blood toxicogenomics reveals potential biomarkers for management of idiosyncratic drug-induced liver injury
Source: Front Genet. 2025 Mar 25;16:1524433. doi: 10.3389/fgene.2025.1524433 (PMC11975945; doi:10.3389/fgene.2025.1524433)
Supplement: Supplementary file 3 [file DataSheet1.docx]

Supplementary Material

# Supplementary Data

Supplemental Tables 1 - 18 (S1- S18) can be viewed in the Supplementary Excel data sheets (Called Table 1 and Table 2).

# Supplementary Figures

**Supplemental Figure 1:** Correlation between CIBERSORTx predicted and clinically observed lymphocyte fractions were determined using Pearson’s r in n=37 high causality IDILI subjects who had a blood collection for % lymphocytes measurement taken within one day of PAXgene tube blood collection for RNA-seq.
